# Effects of Dihydrotanshinone I on Proliferation and Invasiveness of Paclitaxel-Resistant Anaplastic Thyroid Cancer Cells

**DOI:** 10.3390/ijms22158083

**Published:** 2021-07-28

**Authors:** Lorenzo Allegri, Francesca Capriglione, Valentina Maggisano, Giuseppe Damante, Federica Baldan

**Affiliations:** 1 Institute of Medical Genetics, Academic Hospital of Udine, Azienda Sanitaria Universitaria Integrata di Udine, 33100 Udine, Italy; allegri.lorenzo@spes.uniud.it; 2 Department of Health Sciences, University of Catanzaro ‘Magna Graecia’, 88100 Catanzaro, Italy; francesca.capriglione@studenti.unicz.it (F.C.); vmaggisano@unicz.it (V.M.); 3 Department of Medicine, University of Udine, Via Chiusaforte, 33100 Udine, Italy; federica.baldan@uniud.it

**Keywords:** anaplastic thyroid cancer, paclitaxel, dihydrotanshinone, drug resistance

## Abstract

ATC is a very rare, but extremely aggressive form of thyroid malignancy, responsible for the highest mortality rate registered for thyroid cancer. In patients without known genetic aberrations, the current treatment is still represented by palliative surgery and systemic mono- or combined chemotherapy, which is often not fully effective for the appearance of drug resistance. Comprehension of the mechanisms involved in the development of the resistance is therefore an urgent issue to suggest novel therapeutic approaches for this very aggressive malignancy. In this study, we created a model of anaplastic thyroid cancer (ATC) cells resistant to paclitaxel and investigated the characteristics of these cells by analyzing the profile of gene expression and comparing it with that of paclitaxel-sensitive original ATC cell lines. In addition, we evaluated the effects of Dihydrotanshinone I (DHT) on the viability and invasiveness of paclitaxel-resistant cells. ATC paclitaxel-resistant cells highlighted an overexpression of ABCB1 and a hyper-activation of the NF-κB compared to sensitive cells. DHT treatment resulted in a reduction of viability and clonogenic ability of resistant cells. Moreover, DHT induces a decrement of NF-κB activity in SW1736-PTX and 8505C-PTX cells. In conclusion, to the best of our knowledge, the results of the present study are the first to demonstrate the antitumor effects of DHT on ATC cells resistant to Paclitaxel in vitro.

## 1. Introduction

Thyroid cancer (TC) is the most common endocrine carcinoma, accounting for 1–2% of cancer cases worldwide [1]. Most thyroid carcinomas derive from follicular cells and are classified according to their differentiation levels in differentiated thyroid cancer, including papillary thyroid cancer (PTC) and follicular thyroid cancer (FTC) and in poorly differentiated thyroid cancer or anaplastic thyroid cancer (ATC) [1]. While PTC and FTC, which account for more than 95% of tumors, are mostly responsive to the current treatment based on surgery and following radioiodine therapy, resulting in an overall survival rate of over 90% within 10 years, ATCs are a very rare, but extremely aggressive form of thyroid malignancy. They are mostly diagnosed as stage IV disease [1], often are not resectable, and do not respond to radioactive iodine ablation for their undifferentiated phenotype due to the loss of thyroid-specific gene expression [2]. For these reasons and considering its extremely fast-growing and aggressive nature, ATC, although representing less than 2% of all thyroid malignancies, is responsible for 20–50% of thyroid cancer mortality [3].

The current management of ATC has strongly been conditioned by the advent of the molecular testing of thyroid tumors which permits to target the known genetic mutations [4] with the new molecular drugs, mainly inhibitors of tyrosine kinases [5,6]. However, for the tumors resistant to these new drugs and those without known detectable genetic aberrations, the current treatment is still represented by palliative surgery and systemic chemotherapy, the latter consisting in taxane monotherapy or combined with carboplatin or anthracyclines [7]. Unfortunately, even the combined drug treatments easily induce drug resistance and, in the absence of further reliable therapy, patients with ATCs are destined to shortly die due to unlimited local growth and distal metastases dissemination [6,7]. Comprehension of the mechanisms involved in the development of drug resistance has become therefore urgent for improving the prognosis of this very aggressive malignancy. In this context, a pivotal role in promoting cancer resistance to chemotherapy is played by Nuclear factor-kappa B (NF-κB), a transcription factor that regulates the expression of various genes that are crucial for cancer development and progression [8,9]. The role of NF-κB in thyroid cancer has extensively been investigated, especially in ATC suggesting a major involvement in thyroid tumor genesis and progression [10,11]. Interestingly, inhibition of NF-κB by ectopic expression of a super-repressor form of IκBα in an ATC-derived cell line leads to an increased susceptibility to chemotherapeutic drug-induced apoptosis, and the block of the oncogenic activity [11]. Bringing all these data together and considering the influence of NF-κB on the expression of the multidrug resistance gene (*MDR1*), this transcription factor may be also considered as involved in the development of drug resistance in ATCs.

The overexpression of elements of MDR is a well-established cause of therapeutic failure in human cancer. In MDR cells, a key role is played by ATP binding cassette (ABC) transporters, integral membrane pumps responsible for the efflux of drugs from tumor cells [12]. ATP binding cassette subfamily B member 1 (ABCB1), encoded by *MDR1*, the most typical ABC transporter, expels non-polar compounds from the cytosolic side of the membrane to the extracellular space. In the literature, it is well described that ABCB1 overexpression produces MDR in different cancer cells inducing the decrease in the intracellular concentration of ABCB1 substrates, such as taxanes [13,14]. Numerous in vitro and in vivo studies have stated that the overexpression of ABCB1 is involved in mediating resistance to PTX and docetaxel in various types of cancers [15,16]. ATCs exhibit over- and co-expression of different ABC transporters, resulting in amplification of the resistance to a single agent or in multidrug resistance to different compounds [17]. Moreover, inhibition of ABCB1 restores the sensitivity to doxorubicin in a cancer stem cells-rich ATC cell-line as demonstrated by Zheng et al. [18]. These findings suggest that ABCB1 plays a key role in chemotherapy resistance development in ATC.

In recent years, phytochemicals have taken a leading role in the search for new drugs capable of fighting cancer. Indeed, many plants extracted compounds have attracted particular attention as agents provided with anticancer activity and tested in preclinical models of several malignancies [19,20]. Among the phytochemicals tested against ATC cancer cells [21,22,23], particular attention has been focused on 15,16-dihydrotanshinone I (DHT), a tanshinone extracted from *Salvia miltiorrhiza Bunge* (Danshen), one of the most frequently prescribed herbs in traditional Chinese medicine [24]. DHT has not only shown promising anticancer effects in numerous in vitro models of many types of cancer, including ATC [25,26,27], but has also been tested in animal models, confirming its ability to inhibit tumor growth with no adverse effects on healthy tissues [28,29]. Moreover, Hu et al. demonstrated that DHT plays an important role in circumventing colon cancer cell resistance by down-regulating ABCB1 expression and inhibiting its ATPase activity, thereby increasing intracellular accumulation of the transporter substrate anticancer drugs [30]. Altogether, these findings indicate that DHT is a candidate that may be tested as a novel anticancer agent in ATC treatment.

In this study, we created a model of anaplastic thyroid cancer cells resistant to paclitaxel, to investigate the characteristics of these cells by analyzing the profile of gene expression and comparing it with that of paclitaxel-sensitive original ATC cell lines. In addition, we evaluated the effects of Dihydrotanshinone I on the viability and invasiveness of paclitaxel-resistant cells.

## 2. Results

### 2.1. Generation of Paclitaxel-Resistant Anaplastic Thyroid Cancer Cells and Paclitaxel Effects on Cell Viability

**Figure 1:**
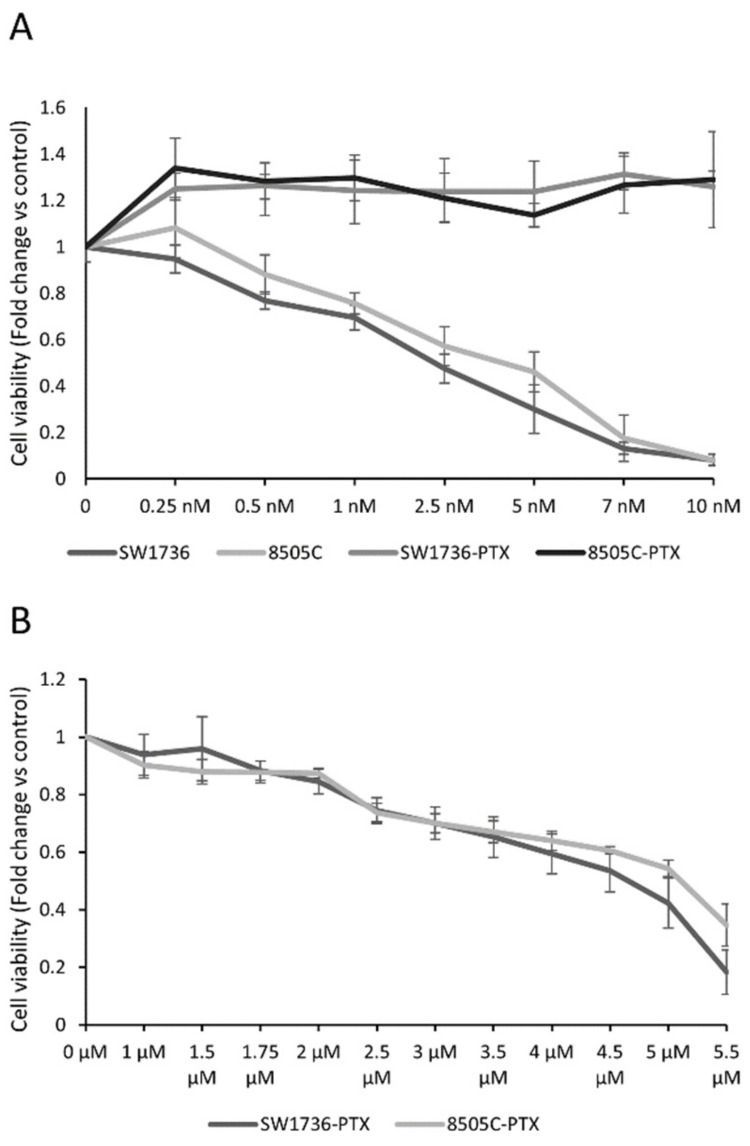
Effects of paclitaxel in ATC cell line cell viability. (**A**) SW1736 and 8505C parental and paclitaxel-resistant (SW1736-PTX and 8505C-PTX) cells were treated with paclitaxel at different doses (rising from 0.25 nM to 10 nM) or vehicle (DMSO) for 72 h and cell viability was assessed by MTT assay. (**B**) SW1736 and 8505C paclitaxel-resistant cells were treated with paclitaxel at different doses (rising from 1 μM to 10 μM) or vehicle (DMSO) for 72 h and cell viability was assessed by MTT assay. Each point represents the mean of six measurements. *n* = 6. *p* values are enlisted in Supplementary Tables S1 and S2.

We used ATC cell lines SW1736 and 8505C to obtain the two parental cell lines resistant to paclitaxel (SW1736-PTX and 8505C-PTX) by the administration of the drug at increasing doses. In the first set of experiments, we evaluated the response to PTX in both wild-type (wt) and resistant ATC cell lines. To test the effects on cell viability of several doses of PTX, we performed an MTT assay after treating ATC cells for 72 h. As shown in Figure 1A, PTX treatment significantly reduced the viability in both wt ATC cell lines when used at a concentration equal or greater than 0.5 nM. The EC50 was 3.2 ± 0.57 nM in SW1736 and 4.3 ± 0.21 nM in 8505C. At variance, resistant ATC cell SW1736-PTX and 8505C-PTX were not sensitive to these low doses, and for this reason, we tested their viability using higher PTX concentrations. After 72 h PTX treatment, the viability in both PTX-resistant cell lines was significantly reduced after administration of PTX concentration higher than 1.75 µM and EC50 shifted to 4.2 ± 0.2 µM and 4.9 ± 0.3 µM in SW1736-PTX and 8505C-PTX respectively (Figure 1B).

### 2.2. ATC and ATC-PTX Cell Lines Gene Expression Analysis

**Figure 2:**
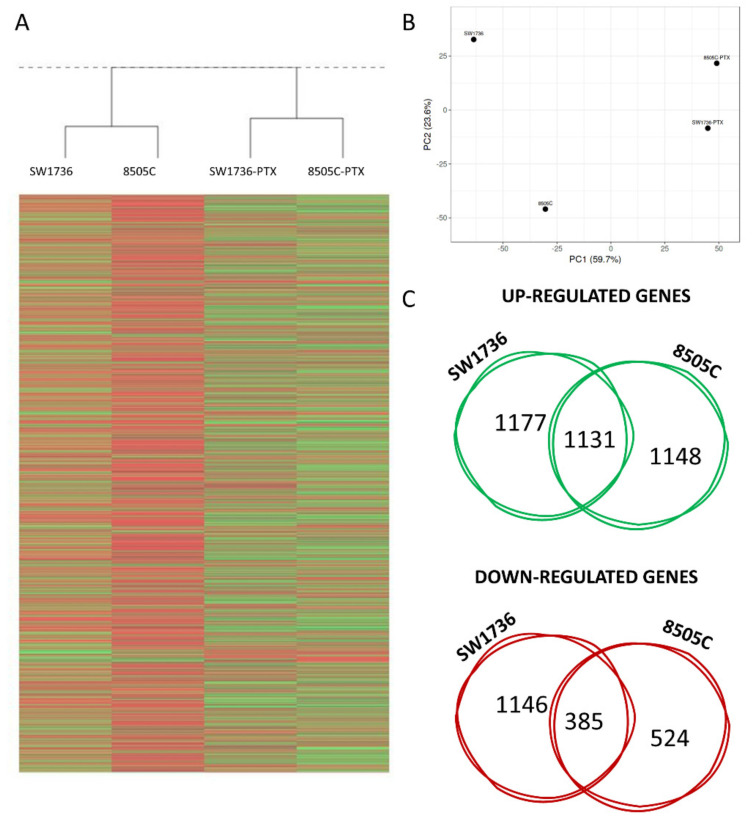
Paclitaxel-resistance effects on gene expression. (**A**) Heat maps showing the hierarchical clustering of mRNA in SW1736 and 8505C cells and in SW1736-PTX and 8505C-PTX cell lines. Results are showed as Fold Change (Log2). (**B**) Principal Component Analysis (PCA) results. Each point represents an RNA-Seq sample. Samples with similar gene expression profiles are clustered together. (**C**) Venn diagrams represented the comparison of up-regulated, down-regulated between SW1736-PTX and 8505C-PTX cell lines after RNA-seq data analysis. Within the intersection of the circles are indicated the shared modified genes between the two cell lines.

To identify the molecular mechanism behind the acquisition of resistance to PTX, we performed a high-throughput RNA sequencing analysis on SW1736 and 8505C cells and on SW1736-PTX and 8505C-PTX cells. To assess the gene expression changes induced by the acquisition of the resistance, a comparison between wild-type and resistant cells was performed. Heat maps representing each analyzed condition are shown in Figure 2A. Expression of 13,919 and 13,386 genes was detected in SW1736 and 8505C cells, respectively. After filtering low quantity reads, approximately 3800 genes were differentially expressed between wild type and resistant SW1736 cells, while in 8505C cells, the differentially expressed genes were ∼3200 (at a log2 fold change > 1.5). Among them, 2308 genes resulted significantly up-regulated and 1531 were down-regulated in SW1736-PTX cells compared to wt SW1736. In 8505C cells, 2279 genes were up-regulated and 909 were down-regulated in response to resistance acquisition. The top 10 up- and down-regulated genes for each cell line are enlisted in Table 1 and Table 2. The principal component analysis (PCA) revealed a clear separation of SW1736 and 8505C wild-type cells and resistant cells. PCA resulted in a separation of wild type and PTX resistant samples along the first principal component (PC1) with 59.7% explained variance, and 23.6% explained variance along the second principal component (PC2) (Figure 2B). Comparison of the enriched genes in both cell lines showed that 1516 genes were commonly altered in the two ATC-PTX cell lines.

**Figure 3:**
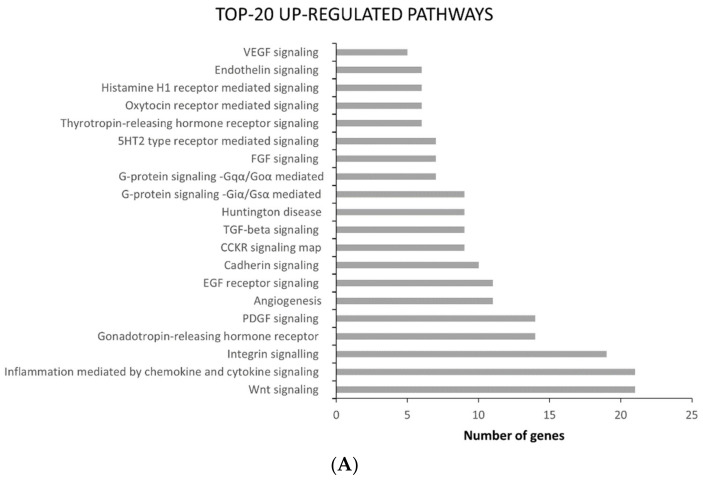

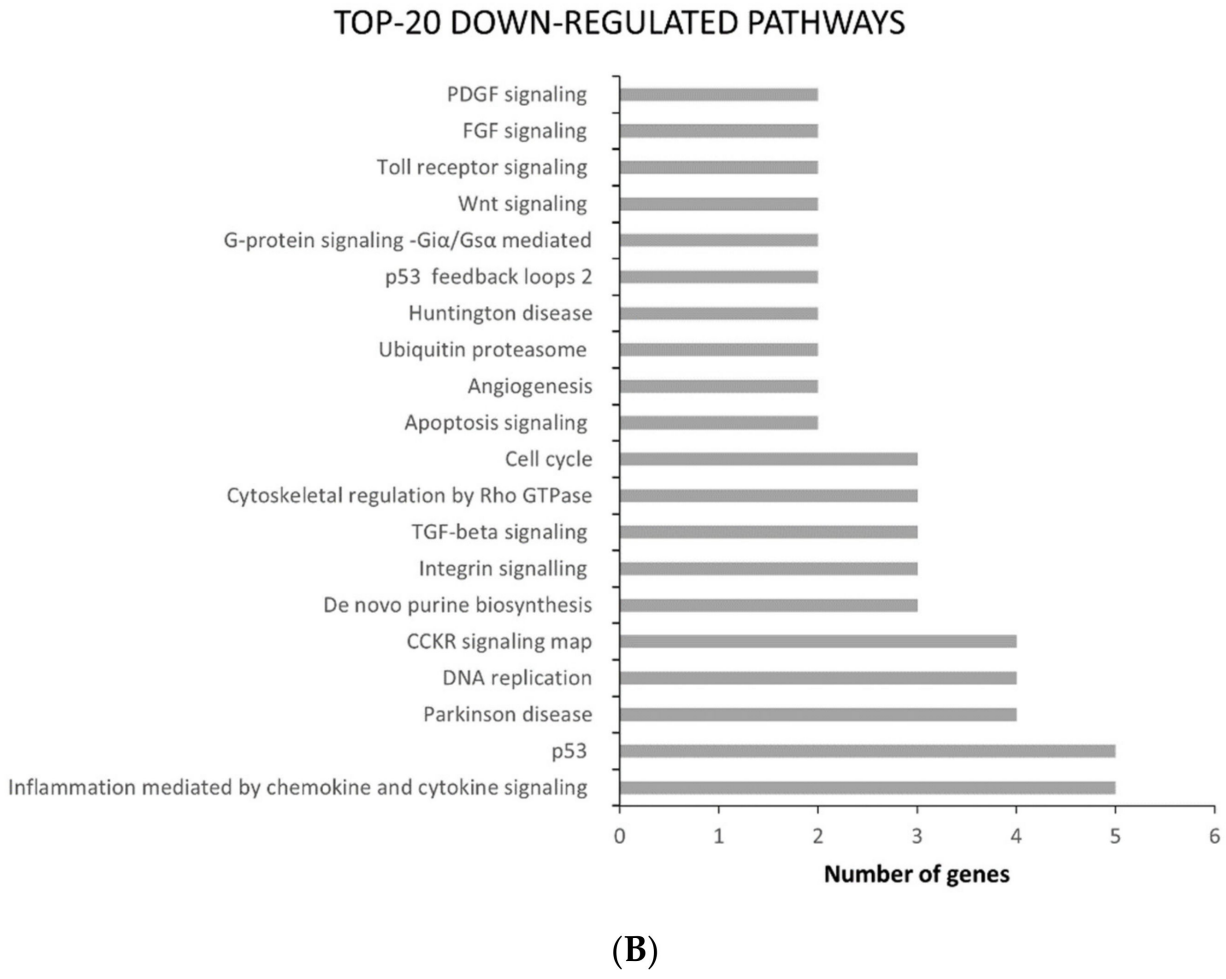
Top 20 deregulated pathways. (**A**) Top 20 common and up-regulated pathways in SW1736-PTX and 8505C-PTX cells. (**B**) Top 20 common down-regulated pathways in SW1736-PTX and 8505C-PTX. Pathway analysis was performed using the Panther classification system.

In Figure 2C, Venn diagrams show that 1131 genes were commonly up-regulated and 385 were down-regulated. The common differentially expressed genes were then subjected to ontology-based Pathway Analysis in order to outline which pathways were mostly affected by Paclitaxel resistance acquisition. Various signaling cascades turned out to be modified in ATC-PTX cells: inflammation mediated by chemokine and cytokine signaling and integrin signaling proved out to be among the most deregulated pathways, since numerous genes involved in these pathways were both up- and down-regulated (Figure 3).

In order to assess the transcription factors most involved in the gene expression deregulation due to the acquisition of paclitaxel resistance, the database TRRUST [31] was queried. Table 3 and Table 4 enlist the top 10 transcription factors involved in the common genes up-regulation and down-regulation, respectively. Among them, SP1, NF-kB and TP53 appear to be strongly involved in both inducing and repressing gene expression of commonly deregulated genes in ATC-PTX cells.

### 2.3. Activation of STAT 3 and ERK in Wild Type and Paclitaxel-Resistant ATC Cells

**Figure 4:**
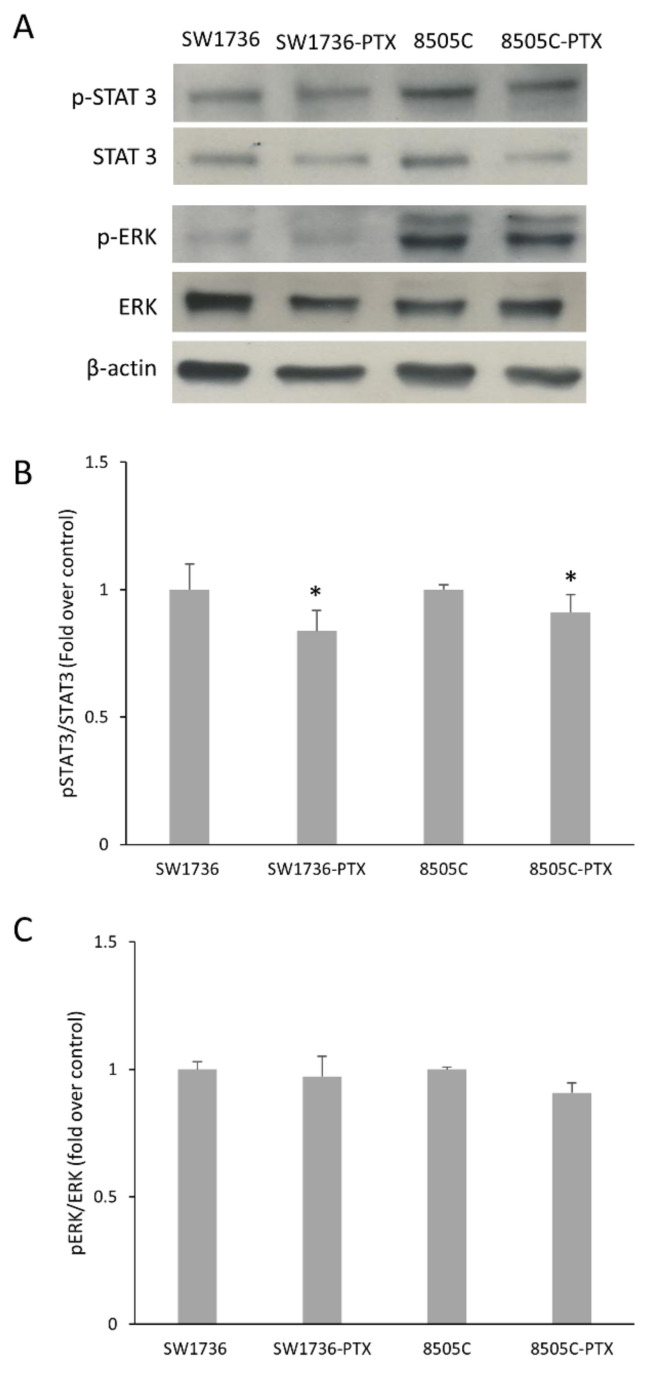
Expression levels of STAT 3 and ERK in wild type and Paclitaxel-resistant ATC cells. (**A**) Immunoblot analysis of phosphorylated ERK (p-ERK) and phosphorylated STAT 3 (p STAT3), and total form of the enzymes (ERK, STAT3), was performed by Western blotting in parental and Paclitaxel-resistant (-PTX) SW1736 and 8505C cells. β-actin was used as loading control. *n* = 3. (**B**) Densitometric analysis of p-ERK/ERK. *n* = 2. Panel (**C**) Densitometric analysis of p-STAT3/STAT3. *n* = 3. Values are expressed as fold over control. Statistical analysis was performed using the Tukey–Kramer multiple comparisons test. * *p* < 0.05 vs. control.

To investigate the involvement of signal transduction pathways activated by the most frequent genetic alterations detected in ATC cells in the cells with acquired resistance to PXT, we performed an immunoblot analysis to measure the expression levels of phosphorylated STAT3 and ERK, markers of activation of JAK/STAT and ERK/MAPK pathways respectively. As shown in Figure 4, we observed a slight decrease in phospho-STAT 3 protein expression levels in SW1736 and 8505C paclitaxel-resistant cells, compared to the parental cells. In the same experimental conditions, no variation of phospho-ERK levels was observed.

### 2.4. Effects of DHT on Cell Viability and Cell Colony Formation Ability in ATC-PTX Cell Lines

**Figure 5:**
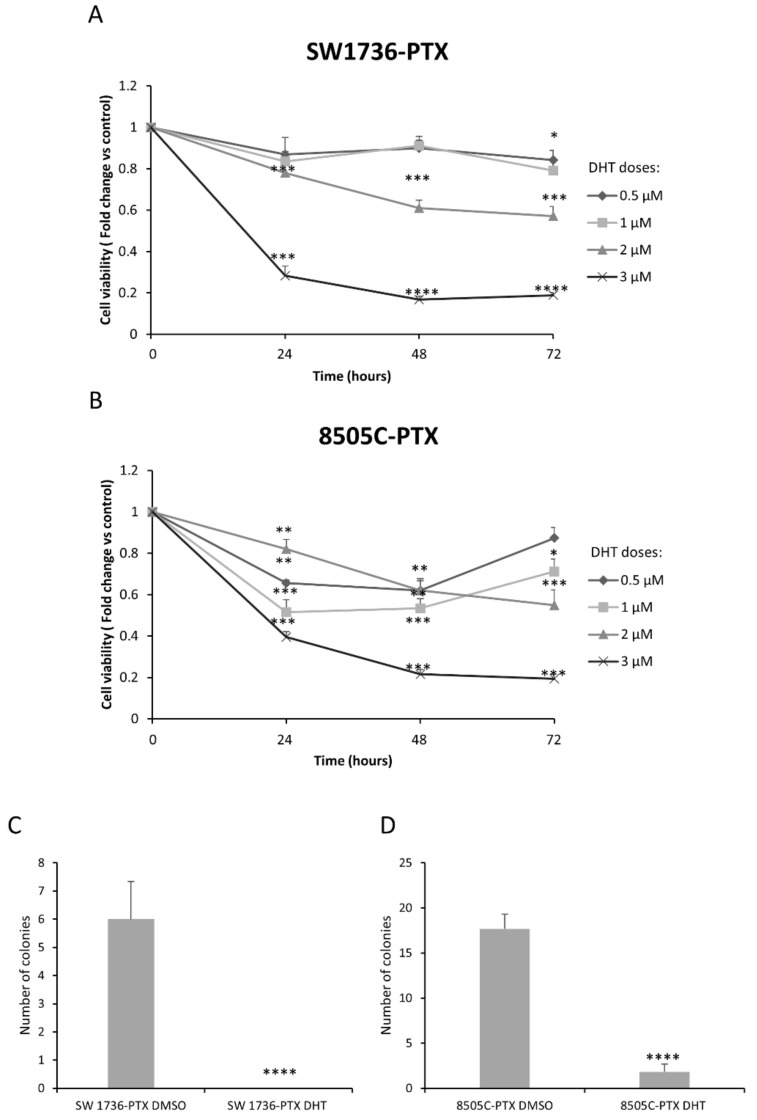
DHT effects in paclitaxel-resistant ATC cells. Panel (**A**,**B**): SW1736-PTX (**A**) and 8505C-PTX (**B**) cells were treated with DHT at different doses (rising from 0.5 μM to 3 μM) or vehicle (DMSO) for 24, 48 and 72 h and cell viability was assessed by MTT assay. Panel (**C**,**D**): SW1736-PTX and 8505C-PTX cells were treated with DHT 2.5 μM and their ability of forming colonies was evaluated by soft agar assay. Representative bar chart of the number of colonies after 21 days in SW1736-PTX (**C**) and 8505-PTX (**D**) cells. *n* = 3. * *p* < 0.05, ** *p* < 0.01, *** *p* < 0.001, **** *p* < 0.0001.

In order to assess the effects of DHT on the viability of PTX-resistant ATC cells, we carried out an MTT assay on SW1736-PTX and 8505C-PTX after treatment with increasing DHT doses for 24, 48 or 72 h. In SW1736-PCTX the two highest doses (2 and 3 µM) reduced cell viability after 24, 48, and 72 h treatment (Figure 5A); conversely, in 8505C cells, all doses tested were able to significantly reduce the cell viability at each time point (Figure 5B). Based on these data, the 2.5 µM dose and 72 h treatment were considered as the common EC50 of the two cell lines and used in the further experiments. To assess the effects of DHT on other characteristics of aggressiveness in the two ATC-PTX cell lines, we assessed its influence on the ability of cells to form colonies in an anchorage-independent way, by performing a soft agar colony formation assay. As shown in Figure 5C,D, there was a significant reduction in the number of colonies when cells were treated with 2.5 µM DHT. In 8505C cells, DHT reduced the number of colonies by approximately 10-fold, and its effects were stronger on SW1736 cells, in which it completely abrogated colony formation ability.

### 2.5. Effects of DHT on Gene and Protein Expression in ATC-PTX Cells

**Figure 6:**
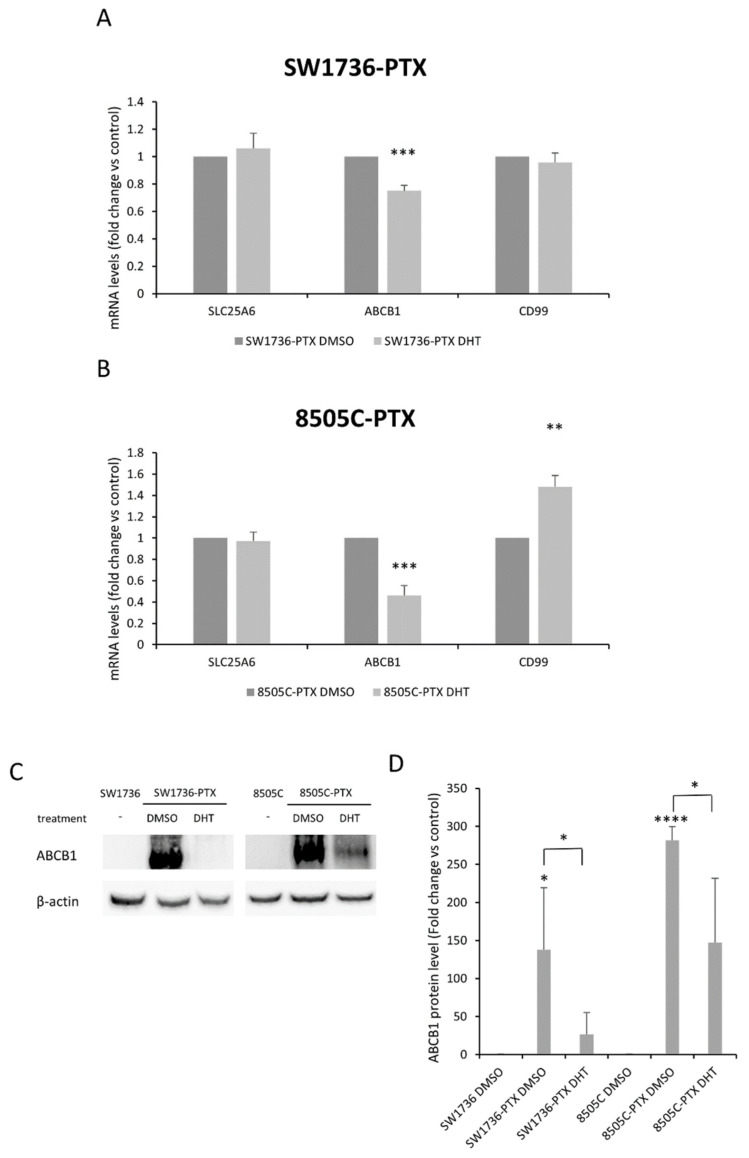
Effects of DHT on gene and protein expression in ATC-PTX cells. SW1736-PTX and 8505C-PTX cells were treated with DHT 2.5 μM or vehicle. Panel (**A**,**B**): Relative expression levels of three commonly deregulated mRNA in SW1739-PTX (**A**) and 8505C-PTX (**B**) following 2.5 µM DHT treatment for 72 h. Data are normalized against β-actin levels. (**C**) ABCB1 protein levels in SW1736-PTX and 8505C-PTX cells treated with DMSO or 2.5 μM DHT for 72 h. (**D**) Densitometric analysis of ABCB1 protein levels in ATC-PTX cells treated with 2.5 μM DHT or DMSO. Data are normalized against β-actin levels. *n* = 3. * *p* < 0.05, ** *p* < 0.01, *** *p* < 0.001, **** *p* < 0.0001.

In order to study the molecular mechanisms underlying the effects of DHT in resistant cells, we focused on commonly up-regulated genes. We analyzed the variation in the gene expression levels following the treatment with DHT of commonly up-regulated genes present in the top 10: *SLC25A6*, *ABCB1* and *CD99*. Among selected commonly up-regulated genes, in SW1736-PTX, DHT induced a significant decrease only in *ABCB1* expression after treatment at 2.5 µM concentration for 72 h (Figure 6A). The same effect on *ABCB1* was also observed in 8505C-PTX cells, in which treatment with DHT also determined an increase in *CD99* expression (Figure 6B).

Since DHT was shown to revert *ABCB1* overexpression in both resistant cell lines, we evaluated its protein levels after treatment with DHT. First, we confirmed its over-expression also at the protein level in both resistant cell lines. Then we assessed the effects of treatment with DHT 2.5 µM for 72 h: immunoblot analysis showed a significant reduction in ABCB1 expression in both SW1736-PTX and 8505C-PTX cells (Figure 6C,D).

### 2.6. Effects of DHT on NF-kB Levels and Activation in ATC-PTX Cell Lines

**Figure 7:**
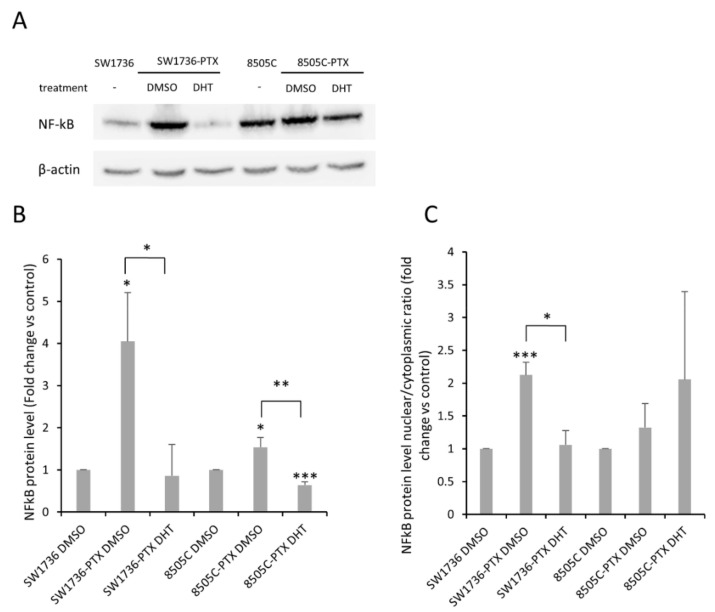
Effects of DHT on NF-κB in ATC and ATC-PTX cells. SW1736 and 8505C cells, both parental and paclitaxel-resistant, were treated with DHT 2.5 μM or vehicle. (**A**) NF-κB protein levels in ATC and ATC-PTX cells treated with DMSO or 2.5 μM DHT for 72 h. (**B**) Densitometric analysis of NF-κB protein levels in ATC and ATC-PTX cells treated with 2.5 μM DHT or DMSO. Data are normalized against β-actin levels. (**C**) Densitometric analysis of NF-KB nuclear/cytoplasmatic protein levels ratio in ATC and ATC-PTX cells treated with 2.5 μM DHT or DMSO. Cytoplasmatic data are normalized against β-actin levels and nuclear data are normalized against Lamin B1. *n* = 3. * *p* < 0.05, ** *p* < 0.01, *** *p* < 0.001.

To better understand the mechanism underlying ABCB1 dysregulation in ATC-PTX cells, in light of the results obtained after high-throughput RNA sequencing analysis, we focused our attention on the transcription factor NF-κB. NF-κB protein expression levels were assessed in ATC cells, and ATC-PTX cells treated with vehicle or 2.5 μM DHT for 72 h. As shown in Figure 7A,B, resistant cells present a strong and significant increase in NF-κB protein expression, and this increment is removed following DHT treatment. However, the total amount of NF-κB within the cell is not a sufficient indicator of its activation status. When its activation pathway is turned on, NF-κB migrates from the cytoplasm into the nucleus, where it can activate the transcription of its target genes. Therefore, in order to obtain a more meaningful result about the role of this transcription factor, we also evaluated the relationship between the nuclear and cytoplasmic forms of NF-κB. Figure 7C shows that this ratio was increased in resistant SW1736 cells compared to the non-resistant parental cell line, indicating a hyper-activation, while DHT treatment again was able to bring NF-κB activation back to normal levels. In 8505C cells, no significant changes in the nucleus–cytoplasm ratio are noted. Taken together, these results indicate that in resistant cells NF-κB is up-regulated and that DHT is able to restore its expression and activation to the levels observed in non-resistant cells.

## 3. Discussion

A combination of surgical removal of the thyroid gland and lymph nodes, with treatment with radioactive iodine (RAI), associated with long-term thyroid hormone suppression is highly efficacious for the majority of thyroid cancers [32]. Conversely, this approach is not effective in the radioiodine refractory DTC and especially in ATC. For these extremely aggressive malignancies, promising effects are being obtained by the use of novel therapeutic approaches [5,6,33], but, in many cases, the resistance to the current treatments is still a fundamental unresolved issue.

In this study, we focused our attention on the molecular mechanism involved in the acquisition of resistance to the treatment with paclitaxel, a taxane still indicated in mono- or multi-therapy for those tumors in which the molecular signature does not suggest the use of personalized treatment with a tyrosine kinase inhibitor [7]. Thus, we first created a model of ATC cells resistant to paclitaxel treatment, using two human cell lines derived from human ATCs and examining their characteristics by the evaluation, firstly, of the gene expression differences between paclitaxel-sensitive parental lines and those in which resistance had developed. Data obtained from the RNA-seq analysis indicate how the acquisition of paclitaxel resistance is associated overall with an increase in the expression of 3456 genes and a down-regulation of 2055 genes. More in detail, the genes commonly up-regulated in the two lines turn out to be 1131, while those commonly down-regulated were 385. SW1736 and 8505C, despite being both cell lines derived from ATC, present important differences at gene expression level, reproducing the high variability shown in the behavior of human ATCs, but these differences are significantly reduced after the acquisition of resistance to Paclitaxel. The similarity in the transcriptional signature between the two resistant cell lines suggests that the mechanism of resistance acquisition may be common and finely regulated rather than the result of random events.

Analyzing the pathways in which commonly up- or down-regulated genes are involved, we found that the one concerning inflammation mediated by cytokine and chemokine signaling turns out to be one of the most altered. *SLC25A6, CD99,* and *ABCB1* were the single genes most commonly up-regulated in the two resistant cell lines. *SLC25A5* is strongly over-expressed in various types of human cancer cells [34], while the role of *CD99* in cancer is more controversial, since it behaves as an oncogene in most cancers, whereas in some types of neoplasia it behaves as a tumor suppressor gene [35]. ABCB1 is a transporter of neutral or positively charged hydrophobic compounds and xenobiotics out of the cell, thereby protecting them from cytotoxicity [36]. ABCC1 has been described as the most highly expressed transporter in ATC [17], and also appears to be overexpressed following treatment with Paclitaxel and Docetaxel in these tumors, thus largely explaining the acquisition of chemoresistance [37,38].

Using as input the up- and down-regulated genes in the two ATC-PTX cell lines, we also identified the transcription factors most involved in the acquisition of resistance to Paclitaxel. Of particular interest was the presence among them of NF-κB as one of the transcription factors most involved, the importance of the NF-κB cascade described in different stages of thyroid cancer [10]. In addition, the JAK/STAT pathway seems to be involved as acquired in the resistant ATC cells in which reduced levels of phosphorylated STAT3 were detected compared to the parental cells. These findings are consistent with the hypothesis of a protective rather than promoting the role of this pathway in the development and progression of thyroid cancer [39], also suggesting a relationship with the acquisition of drug resistance activation.

In previous studies, we and others have already shown that the use of compounds extracted from plants is potentially useful in counteracting the growth of ATC cells in preclinical experimental models [20,21,22,40,41]. In particular, we demonstrated that DHT, a lipophilic abietane diterpene compound extracted from the dried root of *Salvia miltiorrhiza*, has an antitumor action in ATC cell lines, and this action is in part mediated by the regulation of the HuR-MAD2 axis known to be involved in the proliferation, survival and aggressiveness of this tumor [42,43]. In this study, we evaluated the effects of DHT on ATC cell lines resistant to Paclitaxel and found that DHT was effective in reducing cell viability in SW1736-PTX and 8505C-PTX cells with an EC50 absolutely comparable with that observed in non-resistant ATC cells in our previous work [27]. Lee et al. demonstrated that DHT treatment induces a decrease in cell population in the Gl phase, an increase in the S phase cells and apoptosis in erythroleukemia cells resistant to Adriamycin [44]. These results are consistent with those we observed in ATC cells treated with DHT [27], suggesting a similar mechanism underlying the effects observed in ATC-PTX cells.

Furthermore, DHT treatment was able to almost completely reduce the ability to form colonies in an anchorage-independent manner in both cell lines. By analyzing the effects of DHT on the expression of the most relevant up-regulated genes identified after RNA-seq, we found that while the treatment was unable to counteract the increased expression of *SLC25A6* and *CD99* following the acquisition of resistance, DHT significantly reduced *ABCB1* mRNA levels in both ATC-PTX cell lines. Analysis of ABCB1 protein levels also confirmed both its overexpression following the development of resistance and its strong down-regulation following DHT treatment in resistant cells. The effects of DHT on ABCB1 in resistant cells that we observed are in agreement with the results of Hu et al. [30], suggesting that part of the antitumor effects of DHT observed in resistant cells are due to the reduction of ABCB1 expression.

In order to identify a molecular mechanism upstream of ABCB1 down-expression, we shifted our attention to the activation state of NF-κB. A number of NF-kB inhibitors have been demonstrated to induce anti-proliferative and anti-apoptotic effects against thyroid cancer cells, and various anticancer compounds exert their effects by blocking this pathway [10,45,46,47]. Our finding revealed that NF-κB is one of the transcription factors most involved in the acquisition of Paclitaxel resistance in SW1736-PTX and 8505C-PTX cells, which is consistent with the known effect of NF-κB on the increase in ABCB1 expression [9]. By analyzing total protein levels and the ratio of nuclear versus cytoplasmic levels, we also demonstrate that NF-κB appears to be up-regulated in resistant cell lines compared to sensitive ones, whereas DHT treatment results in a reduction of NF-κB activity in SW1736-PTX and 8505C-PTX cells. This finding allows us to hypothesize that inhibition of NF-κB activity may be one of the mechanisms underlying the down-regulation of ABCB1. Considering the wide range of pathways on which DHT is active, other mechanisms of action, such as the regulation of intracellular calcium mobilization already observed by Park et al. [48], STAT-3 and other transduction signaling pathways, could be involved and more in-depth studies are advisable.

In conclusion, to the best of our knowledge, the results of the present study are the first to demonstrate the antitumor effects of DHT on ATC cells resistant to Paclitaxel in vitro. Despite these encouraging results, this study is limited by the lack of validation of the results by using an in vivo model. Therefore, further and more detailed studies on DHT effects on ABCB1 and NF-κB regulation, as well as in vivo investigation of this compound in combination with paclitaxel or other anticancer compounds, are needed in view of testing it in clinical trials.

## 4. Methods

### 4.1. Cell Lines

SW1736 and 8505C cells, derived from human ATC, were cultured in RPMI-1640 medium (Euroclone S.p.A, Milano, Itlay) supplemented with 10% FBS (Gibco; Thermo Fisher Scientific, Inc., Waltham, MA, USA), 2 mM L-glutamine (Euroclone S.p.A) and 50 mg/mL gentamicin (Gibco; Thermo Fisher Scientific, Inc.). Cells were cultured in a humidified incubator (5% CO_2_ and 95% air at 37 °C) (Eppendorf AG, Hamburg, Germany). Both cell lines were validated using short tandem repeat analysis and confirmed to be mycoplasma-free. SW1736 and 8505C cells were treated with DMSO (vehicle; Sigma-Aldrich; Merck KGaA, Darmstadt, Germany), DHT (purity ≥ 99%; Selleck Chemicals, Houston, TX, USA) or Paclitaxel (Selleck Chemicals). The DHT was prepared by dissolving 2 mg of powder in 2 mL of DMSO, resulting in a 3.6 mM stocking solution.

To obtain ATC paclitaxel-resistant cell lines (SW1736-PTX and 8505C-PTX), the two parental cell lines were treated with increasing doses of paclitaxel arising from 1 nM to 1.5 μM for 6 months. Resistance to pacitaxel was considered to be acquired when a 1.5 μM dose resulted in no effect in terms of cell viability. Resistant cells were cultured in RPMI-1640 medium supplemented with 10%, 2 mM L-glutamine, 50 mg/mL gentamicin and paclitaxel 1.5 μM.

### 4.2. Cell Viability

In order to test cell viability, the MTT assay was used. SW1736 and 8505C cells, parental and PTX-resistant, were seeded in 96-well plates (4 × 10^3^ cells/well). The following day, cells were treated with paclitaxel (from 0.25 nM to 10 nM in parental cells and from 1 μM to 5.5 μM in resistant cells), DHT (from 0.5 μM to 3 μM) or vehicle (DMSO) at different concentrations. After 24, 48 or 72 h of incubation, 4 mg/mL MTT (Sigma-Aldrich; Merck KGaA) was added to the cell medium and cells were cultured for a further 4 h in the incubator in the dark. The supernatant was removed, 100 µL/well DMSO (Sigma-Aldrich; Merck KGaA) was added, and the absorbance at 570 nm was measured. All experiments were performed as six technical repeats and cell viability is expressed as the fold-change relative to the control (DMSO-treated cells).

### 4.3. High-Throughput RNA Sequencing and Analysis

RNA was extracted from SW1736 and 8505C cells and from SW1736-PTX and 8505C-PTX cells using a RNeasy Mini kit (Qiagen GmbH, Venlo, Netherlands) according to the manufacturer’s instructions. In total, ~1 μg RNA (RNA integrity number > 7) was used as the starting material for preparation of the library using a Universal Plus mRNA-Seq kit (catalog number 0520-A01; Tecan Group, Ltd., Männedorf, Svizzera) according to the manufacturer’s protocol. RNA samples were quantified, and the quality was assessed using an Agilent 2100 Bioanalyzer RNA assay (Agilent Technologies, Inc., Santa Clara, CA, USA) and the final libraries were checked using both a Qubit 2.0 Fluorometer (Invitrogen; Thermo Fisher Scientific, Inc.) and Agilent Bioanalyzer DNA assay. The library final concentration was 68.7 nM. Libraries (1.4 nM) were then prepared for sequencing using the single-end 75 bp mode on a NextSeq 500 (Illumina, Inc., San Diego, CA, USA). The Bcl2Fastq version 2.20 in the Illumina pipeline was used for processing the raw data (format conversion and de-multiplexing); adapter sequences were masked with Cutadapt version 1.11 [49] from raw fastq data and the ERNE [50] software was used to remove lower quality bases and adapters. Reads were aligned to the reference hg38 genome/transcriptome using STAR software [51]. Finally, assembly and quantitation of full-length transcripts representing multiple spliced variants for each gene locus were performed using the Stringtie tool [52]. For further analysis, we selected effective data for those with FPKM values > 0.5 and log2 fold-change > 1.5 or <−1.5. Raw and processed data are available on the public online repository Gene Expression Omnibus (dataset no. GSE179786).

### 4.4. Gene Expression Assay

A total of 500 ng total RNA from SW1736 and 8505, parental or resistant, cells was extracted as described above, and reverse transcribed to cDNA using random hexaprimers and SuperScript III reverse transcriptase (Thermo Fisher Scientific, Inc.). Quantitative PCR was performed using PowerUP Sybr green master mix (Thermo Fisher Scientific, Inc.) on the QuantStudio3 system (Applied Biosystems; Thermo Fisher Scientific, Inc.). The QuantStudio Design and Analysis software v1.5.0 (Applied Biosystems; Thermo Fisher Scientific, Inc.), was used to calculate mRNA levels with the 2^−∆∆Cq^ method and ß-actin was used as reference. All experiments were performed in triplicate. Oligonucleotide primers were purchased from Sigma-Aldrich and the sequences of the primers are available upon request.

### 4.5. Western Blot Analysis

SW1736 and 8505C parental or PTX-resistant cells were untreated or treated with 2.5 μM DHT or DMSO. After 72 h treatment, total proteins were extracted by using a cell scraper and lysis buffer (50 mM Tris HCl, pH 8; 120 mM NaCl; 5 mM EDTA; 1% Triton; 1% NP40; 1 mM DTT), supplemented with phenyl-methylsulphonyl fluoride and protease inhibitors. Lysates were centrifuged at 13,000× *g* for 10 min at 4 °C, and supernatants were quantified using a Bradford assay. For nuclear and cytoplasmic protein extraction, SW1736 and 8505C parental or PTX-resistant cells were lysed by using two buffer with different ionic force: B1 (HEPES pH 7.9 10 mM, KCl 10 mM, MgCl_2_ 0.1 mM, EDTA pH 8 0.1 mM) for cytoplasmic proteins extraction and B2 (HEPES pH 7.9 20 mM, NaCl 420 mM, MgCl_2_ 1.5 mM, EDTA pH 8 0.1 mM, glicerolo 5%) for nuclear proteins extraction, as previously described [53].

To evaluate pSTAT3, STAT3, pERK and ERK protein levels, 30 μg of total proteins were run on 12% SDS PAGE gel, transferred to PVDF membranes (VWR, Milan, Italy), blocked with TTBS/milk (TBS, 1% Tween 20 and 5% non-fat dry milk) and incubated overnight with anti-STAT 3 antibody diluted 1:1000 or anti-phospho-STAT 3 antibody diluted 1:200 (Cell Signaling, Euroclone, Milan, Italy), anti-ERK antibody diluted 1:1000 or anti-phospho-ERK antibody diluted 1:500 (Santa Cruz Biotechnology, Dallas, TX, USA) and anti-β-actin antibody diluted 1:10,000 (Merck Serono, Rome, Italy). The membranes were incubated with horseradish peroxidase-conjugated antibody (Transduction Laboratories, Lexington, KY, USA) in TTBS/milk. Western blot detection system ECL Plus (Perkin Elmer, Monza, Italy) was used to visualize the proteins.

To evaluate ABCB1 and NF-kB protein levels, 30 µg protein was loaded per lane on a 10% SDS gel, resolved using SDS-PAGE, then transferred to nitrocellulose membranes (GE Healthcare, Chicago, IL, USA). The membranes were blocked at room temperature for 1 h using PBS-milk (PBS; 0.1% Tween-20; 5% non-fat dry milk). The membranes were then incubated overnight at 4 °C with rabbit anti-actin antibody (1:1000; Sigma-Aldrich; Merck KGaA), rabbit anti-lamin B1 (1:1000; Abcam, Cambridge, UK), mouse monoclonal anti-ABCB1 (1:1000; ThermoFisher), rabbit monoclonal anti-NF-kB (1:1000; Active Motif). The following day, the membranes were incubated with peroxidase-conjugated anti-rabbit or anti-mouse IgG secondary antibody (both 1:4000; cat. nos. A6154 and A9044, respectively; both Sigma-Aldrich; Merck KGaA) for 2 h at room temperature. A UVITEC Alliance LD (Uvitec Ltd., Cambridge, UK) Western blot detection system with SuperSignal Technology reagent (Thermo Fisher Scientific, Inc) and the Alliance 1D Max software (Uvitec Ltd.) was used to visualize the signals.

### 4.6. Soft Agar Assay 

The clonogenic ability of the SW1736, 8505C, SW1736-PTX and 8505C-PTX cells was evaluated using a soft agar assay, as previously described [40]. Briefly, after 48 h of treatment, cells were collected, and 1 × 10^4^ cells were suspended in 4 mL complete medium containing 0.25% agarose (Sigma-Aldrich), then seeded to the top of a 1% agarose complete medium layer in 6-cm plates. The colonies were counted by eye in four different fields, under a Leica DMI-600B inverted microscope (Leica Microsystems Ltd., Wetzlar, Germany). Data are representative of three independent experiments.

### 4.7. Statistical Analysis

Data are presented as the mean ± standard deviation. All results were analyzed using the unpaired Student’s *t*-test or the Tukey–Kramer multiple comparisons test in GraphPad Prism version 6 (GraphPad Software, Inc., San Diego, CA, USA). *p* < 0.05 was considered to indicate a statistically significant difference.

## Figures and Tables

**Table 1:** Top 10 up- and down-regulated genes in SW1736-PTX cells.

Top-10 Up-Regulated Genes			Top-10 Down-Regulated Genes		
Gene ID	SW1736 FPKM	SW1736-PTX FPKM	Gene ID	SW1736 FPKM	SW1736-PTX FPKM
*SLC25A6*	0	1068.014282	*CRIP1*	121.2277	0
*CD99*	0	438.171265	*INHBA*	120.7393	0
*SFRP1*	0	353.209839	*G0S2*	110.1006	0
*ABCB1*	0	302.292755	*TMEM200A*	80.16118	0
*ASS1*	0	272.908447	*MT1L*	70.32181	0
*MMP1*	0	253.921478	*CDCA7L*	64.91097	0
*KISS1*	0	227.140152	*PEG10*	59.8796	0
*CD24*	0	161.055496	*CDH13*	56.48613	0
*LAMA5*	0	128.902344	*GNG11*	48.80974	0
*EPB41L3*	0	111.837128	*LIF*	47.06105	0

**Table 2:** Top 10 up- and down-regulated genes in 8505C-PTX cells.

Top-10 Up-Regulated Genes			Top-10 Down-Regulated Genes		
Gene ID	8505C FPKM	8505C-PTX FPKM	Gene ID	8505C FPKM	8505C-PTX FPKM
*SLC25A6*	0	906.9684	*PSMD5*	4.736901	0
*ABCB1*	0	497.1158	*STEAP1*	4.928442	0
*CD99*	0	344.5431	*VEPH1*	5.764932	0
*NUPR1*	0	117.2764	*NLGN4X*	6.481269	0
*ASMTL*	0	44.84652	*IL1RL1*	7.034636	0
*ERV3-1*	0	42.39337	*RPL36A-HNRNPH2*	7.240747	0
*ALPPL2*	0	36.63164	*CDCP1*	8.863821	0
*WFDC21P*	0	33.00259	*ZNF702P*	8.940498	0
*ZNF117*	0	31.36288	*UCHL1*	24.07678	0
*PIEZO2*	0	28.97086	*CBWD5*	67.27538	0

**Table 3:** Regulators of common up-regulated genes.

Key TF	Description	# of Overlapped Genes
SP1	Sp1 transcription factor	10
NFKB1	nuclear factor of kappa light polypeptide gene enhancer in B-cells 1	8
SPI1	spleen focus forming virus (SFFV) proviral integration oncogene spi1	5
WT1	Wilms tumor 1	4
STAT1	signal transducer and activator of transcription 1, 91 kDa	4
JUN	jun proto-oncogene	4
TP53	tumor protein p53	4
RFXANK	regulatory factor X-associated ankyrin-containing protein	3
RFXAP	regulatory factor X-associated protein	3
RFX5	regulatory factor X, 5	3

TF: transcriptor factor.

**Table 4:** Regulators of common down-regulated genes.

Key TF	Description	# of Overlapped Genes
E2F1	E2F transcription factor 1	12
SP1	Sp1 transcription factor	10
TP53	tumor protein p53	7
MYCN	v-myc myelocytomatosis viral related oncogene,	5
MYC	v-myc myelocytomatosis viral oncogene homolog	5
NFKB1	nuclear factor of kappa light polypeptide gene enhancer in B-cells 1	5
SRF	serum response factor	4
BRCA1	breast cancer 1, early onset	4
TFDP1	transcription factor Dp-1	3
RB1	retinoblastoma 1	3

TF: transcriptor factor.

## Data Availability

RNA-seq raw and processed data are available on the public online repository Gene Expression Omnibus (dataset no. GSE179786).

## Supplementary Materials

The following are available online at https://www.mdpi.com/article/10.3390/ijms22158083/s1.
