# Supplementary material for: Effects of Dihydrotanshinone I on Proliferation and Invasiveness of Paclitaxel-Resistant Anaplastic Thyroid Cancer Cells
Source: Int J Mol Sci. 2021 Jul 28;22(15):8083. doi: 10.3390/ijms22158083 (PMC8347033; doi:10.3390/ijms22158083)
Supplement: Supplementary file 1 [file ijms-1312349-supplementary.pdf › ijms-1312349-supplementary.pdf]

Supplementary Table S1. Figure 1, Panel A pValues

Cell line	0.25 nM	0.5 nM	1 nM	2.5 nM	5 nM	7 nM	10 nM
SW1736	ns	0,0004	0,0005	0,0001	0,0003	<0,0001	<0,0001
8505C	ns	ns	0,0007	0,0009	0,0004	0,0001	<0,0001
SW1736-PTX	0,002	0,0004	0,032	0,046	0,0375	0,004	0,0017
8505C-PTX	0,008	0,0023	0,0051	0,02	0,0108	0,0159	ns

Supplementary Table S2. Figure 1, panel B pValues

Cell line	1 µM	1,5 µM	1,75 µM	2 µM	2,5 µM	3 µM	3,5 µM	4 µM	4,5 µM	5 µM	5,5 µM
SW1736	0,0528	0,378	>0,0001	>0,0001	>0,0001	>0,0001	>0,0001	>0,0001	>0,0001	>0,0001	>0,0001
8505C	0,0004	>0,0001	>0,0001	>0,0001	>0,0001	>0,0001	>0,0001	>0,0001	>0,0001	>0,0001	>0,0001
